# Enhancing the Distinguishability of Minor Fluctuations in Time Series Classification Using Graph Representation: The MFSI-TSC Framework

**DOI:** 10.3390/s25154672

**Published:** 2025-07-29

**Authors:** He Nai, Chunlei Zhang, Xianjun Hu

**Affiliations:** College of Electronic Engineering, Naval University of Engineering, 717 Jiefang Avenue, Wuhan 430030, China; nahan1992@126.com (H.N.); xianjunh1985@outlook.com (X.H.)

**Keywords:** time series classification, minor fluctuations, identification, graph, classification accuracy, computational efficiency

## Abstract

In industrial systems, sensors often classify collected time series data for incipient fault diagnosis. However, time series data from sensors during the initial stages of a fault often exhibits minor fluctuation characteristics. Existing time series classification (TSC) methods struggle to achieve high classification accuracy when these minor fluctuations serve as the primary distinguishing feature. This limitation arises because the low-amplitude variations of these fluctuations, compared with trends, lead the classifier to prioritize and learn trend features while ignoring the minor fluctuations crucial for accurate classification. To address this challenge, this paper proposes a novel graph-based time series classification framework, termed MFSI-TSC. MFSI-TSC first extracts the trend component of the raw time series. Subsequently, both the trend series and the raw series are represented as graphs by extracting the “visible relationship” of the series. By performing a subtraction operation between these graphs, the framework isolates the differential information arising from the minor fluctuations. The subtracted graph effectively captures minor fluctuations by highlighting topological variations, thereby making them more distinguishable. Furthermore, the framework incorporates optimizations to reduce computational complexity, facilitating its deployment in resource-constrained sensor systems. Finally, empirical evaluation of MFSI-TSC on both real-world and publicly available datasets demonstrates its effectiveness. Compared with ten benchmark methods, MFSI-TSC exhibits both high accuracy and computational efficiency, making it more suitable for deployment in sensor systems to complete incipient fault detection tasks.

## 1. Introduction

Due to incipient faults [1], often characterized by low amplitude in time series data [2], they are frequently ignored by sensor systems, which may lead to significant degradation in system performance over time [3]. In industrial settings [4], the time series classification [5] of sensor data is a prevalent approach for fault diagnosis [6]. Consequently, the development and implementation of accurate and efficient time series classification methods [7] for identifying these incipient faults [8] is of paramount importance for proactively maintaining the optimal performance and reliability of industrial equipment [9,10].

**Figure 1:**
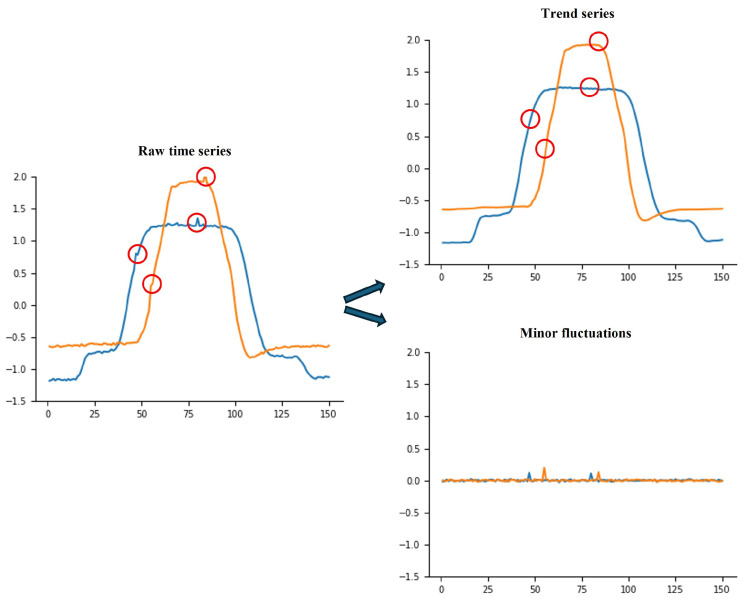
Minor fluctuations in time series. In raw time series, the classification of these time series mainly relies on the minor fluctuations marked with red circles. The amplitude of minor fluctuations is low, leading the classification models to prioritize and learn trend features while ignoring the minor fluctuations crucial for accurate classification.

In the time series data collected by sensors, incipient faults manifest as minor fluctuations in the time series. Existing classification methods often exhibit limited accuracy when applied to time series where minor fluctuations serve as the primary distinguishing characteristic, as shown in Figure 1. While the trends of the two example time series differ, the minor fluctuations, highlighted by red circles, represent the primary distinguishing characteristic for classification. Traditional decomposition methods, such as Wavelet Decomposition (WD) [11] or Empirical Mode Decomposition (EMD) [12], can roughly be employed to separate these minor fluctuations from the trends, resulting in two components: a trend series and a fluctuation series. However, the distinction between the minor fluctuations of the two examples remains minimal, as the amplitude difference between the raw series stems from the disparity in their trend. The limitations in classification accuracy for these types of time series arise from two factors:

(1) The low-amplitude variations inherent in minor fluctuations are often superimposed on a more pronounced trend. Consequently, the classification methods prioritize and learn trend features while ignoring the minor fluctuations that serve as the key basis for classification.
(2) Current methods struggle to differentiate between genuine minor fluctuation features and noise. Attempts to obtain minor fluctuations with existing decomposition methods result in a mixture of relevant features and noise, further leading to a decrease in the accuracy of identifying minor fluctuations.

Time series classification is a prevalent technique for fault detection in sensor systems. Existing classification methods can be grouped into distance-based, series approximation-based, shapelet-based, dictionary-based, transform domain-based, and ensemble learning-based approaches.

Distance-based methods typically rely on calculating the distance between time series. An initial approach used Euclidean Distance (ED) [13], representing the straight-line distance between two points in Euclidean space. However, ED is often unsuitable for time series with phase offsets, as these offsets can inflate the calculated distance, overshadowing minor fluctuation feature differences. The Z-normalized Euclidean distance [14] attempts to mitigate this by normalizing the time series before calculating the ED; however, this normalization can reduce the importance of low-amplitude variations, causing a decrease in classification accuracy. Dynamic Time Warping (DTW) [15] addresses the limitations of ED by allowing for non-linear stretching and compression of the time axis [16], enabling distance calculations between series with unequal length. Unlike direct measures like ED, DTW can accommodate nonlinear time variations within the time series [17]. However, DTW operates directly on the raw amplitude, making it challenging to classify time series when low-amplitude variations are the primary distinguishing feature.

Series approximation-based methods aim to reduce computational complexity by smoothing the time series. Piecewise Aggregate Approximation (PAA) [18] is a common method, dividing the time series into equal-length subsegments and extracting the mean value of each subsegment as a representative feature. Variations of PAA, such as Adaptive Piecewise Constant Approximation (APCA) [19] and Multi-resolution Piecewise Aggregate Approximation (MPAA) [20], employ unequal segmentation to better capture low-amplitude features during the approximation process. Nevertheless, these methods remain approximate and may not be optimal for extracting minor fluctuation features.

Shapelet-based methods represent each class of time series by extracting representative subseries of a specific length, referred to as shapelets [21]. Early shapelet methods enumerated all possible shapelets, resulting in high computational costs and slow performance, particularly with increasing data size. More recent approaches, such as learning time series shapelets, utilize gradient descent methods to learn one or more shapelets through a loss function [22]. Classification is then based on the minimum distance between a given series and the learned shapelets. This approach avoids the need to pre-define a candidate set of subsequences, improving computational efficiency.

Dictionary-based methods represent time series as sequences of symbols, from which words are extracted using a sliding window. Classification is then performed based on the distribution of these words. The Bag-Of-Symbols in Vector Space (BOSS) [23] method leverages the Term Frequency-Inverse Document Frequency (TF-IDF) technique from natural language processing, applying it to Symbolic Fourier Approximation (SFA) [24]. The Bag-Of-Symbols in Vector Space (BOSS) method leverages the Term Frequency-Inverse Document Frequency (TF-IDF) technique from natural language processing, applying it to Symbolic Fourier Approximation (SFA). The BOSS method converts time series into symbol series and utilizes its vector space model for classification. Word Extraction for time Series Classification (WEASEL) [25] is another dictionary-based method that captures words across all sequences by sliding windows of different lengths. WEASEL uses SFA with equal-frequency and equal-depth to record the difference between two adjacent points, which is also captured as a word. Then, the window length, captured words, and their counts are aggregated with a histogram.

Transform domain-based methods transform time series from the time domain to another domain, such as the frequency domain. The Discrete Fourier Transform (DFT) [26] is a foundational technique that decomposes a time series into its frequency components. High-frequency components represent rapid variations, while low-frequency components represent trends. Discrete Wavelet Transform (DWT) [27] extends DFT by incorporating information in time, enabling analysis of how spectral components change over time.

ROCKET (Random Convolutional Kernel Transform) [28] utilizes random convolutional kernels to extract features from time series. The core idea of ROCKET is randomly generating a multitude of convolutional kernels with different parameters. By convolving the convolution kernel with the data to obtain feature vectors. These feature vectors are then combined with a linear classifier to perform the classification task.

Ensemble learning-based methods, such as the Hierarchical Vote Collective of Transformation-based Ensembles (COTE) [29], combine multiple transformation-based methods to improve accuracy and robustness. COTE extracts a diverse set of time series features, including spectral and time-frequency representations. These features are then subjected to various transformations to enhance model diversity and robustness. COTE extracts a diverse set of time series features, including spectral and time-frequency representations. These feature sets are fed into different classifiers, and their individual classifications are combined through voting or weighted averaging to obtain the final classification result. While this type of ensemble learning method can achieve high classification accuracy, it also tends to be computationally complex.

The above classification methods often exhibit limitations in classification when minor fluctuations serve as the primary distinguishing feature within time series. To enhance the classification accuracy of this type of time series, our approach involves representing raw time series in a high-dimensional space. This strategy aims to make minor fluctuations more distinguishable by introducing another dimension of information, thereby facilitating the discrimination of minor amplitude variations within the high-dimensional representation.

Specifically, we extract the “visible relationship” between amplitude values at each pair of time points in the time series and leverage this information to generate a high-dimensional representation incorporating autocorrelation. A similar process is applied to the corresponding trend series. Both the raw series and trend series are thus represented as graphs. By differentiating these graphs, we capture the differences in autocorrelation information resulting from minor fluctuations. Consequently, identifying these difference graphs serves to make these minor fluctuations more distinguishable.

The contributions of this paper are as follows:

(1) We propose a novel time series representation method incorporating autocorrelation information through a concept we term “visible relationship.” This method extracts these relationships between amplitude values at each time step and encodes the time series in a high-dimensional space. By representing the time series as a graph, the low-amplitude variations in minor fluctuations are more distinguishable. Furthermore, we have optimized this representation method to reduce its computational complexity.
(2) We propose a classification framework built upon the representation method, designed to accurately classify time series where minor fluctuations serve as the primary distinguishing features. This framework represents both the raw and trend series as graphs, calculates the difference graph between these representations, and extracts the degrees of nodes in the difference graph to form a degree sequence. This degree sequence contains the high-dimensional difference information induced by minor fluctuations. We demonstrate that utilizing this degree sequence as a feature for classification improves the classification accuracy for time series distinguished by minor fluctuations.

The structure of this article is as follows. Section 2 gives the details of MFSI-TSC, including time series classification problem modeling, the representation method and its optimization, the feature extraction method for time series, and the details of the MFSI-TSC framework. Section 3 provides comprehensive experimental results and discussion. Section 4 concludes the article.

## 2. Materials and Methods

In this section, we introduce our proposed graph representation method for time series and MFSI-TSC framework.

### 2.1. Graph Representation Method for Time Series

To address the challenge posed by the low-amplitude variations of minor fluctuations, this study introduces an additional dimension of information by representing time series within a high-dimensional space. Specifically, both the raw time series and its trend series are represented as graphs, and the topological differences between these graphs are then calculated. By identifying these topological differences, we aim to enhance distinguishability, thereby improving the ability to distinguish minor fluctuations from trends with greater statistical significance.

Trend series are extracted from the raw time series with Wavelet Decomposition (WD) or Empirical Mode Decomposition (EMD), as mathematically formulated in Equation (1) and Equation (2), respectively.

(1) $$f\left(t\right)=\sum_{k=-\infty }^{+\infty }c_{H,k}\phi_{H,k}\left(t\right)+\sum_{h=-\infty }^{h=H}\sum_{k=-\infty }^{+\infty }d_{h,k}\psi_{h,k}\left(t\right)$$

(2) $$f\left(t\right)=\sum_{i=1}^{k}IMF_{i}\left(t\right)+r_{k}\left(t\right)$$

Here, $\phi_{H,k}\left(\cdot \right)$ denotes the scaling function, and $\psi_{h,k}\left(\cdot \right)$ denotes the wavelet function, with $\phi_{H,k}\left(\cdot \right)$ being orthogonal to $\psi_{h,k}\left(\cdot \right)$. $c_{H,k}$ and $d_{h,k}$ represent the scaling and wavelet coefficients, respectively. Consequently, $c_{H,k}\phi_{H,k}\left(t\right)$ captures the low-frequency component of the sequence at scale *H*, while $d_{h,k}\psi_{h,k}\left(t\right)$ represents the corresponding high-frequency components. The low-frequency component represents the trend, and the high-frequency components represent fluctuations in the time series. In Equation (2), $IMF_{i}\left(t\right)$ denotes the *i*-th Intrinsic Mode Function (IMF) of f(t), and $r_{k}\left(t\right)$ denotes the residual component remaining after the extraction of the IMFs from f(t). IMF represents the trend of raw series, while the residual components represent fluctuations in time series.

While EMD offers a powerful, data-adaptive approach to time series decomposition without requiring pre-defined basis functions, it exhibits strong adaptability for analyzing nonlinear and non-stationary signals. However, it is susceptible to noise and prone to mode mixing. WD provides simultaneous time-domain and frequency-domain information, rendering it effective for analyzing signals with consistent patterns. However, the efficacy of WD hinges on selecting an appropriate mother wavelet for the specific characteristics of the time series under analysis.

Subsequently, we introduce a novel autocorrelation measure termed the “visible relationship” to represent the time series in a high-dimensional space. The visible relationship quantifies the interconnectedness between amplitude values at different time points within the time series; it is defined by Equation (3).

(3) $$x_{l}<x_{i}+\left(x_{j}-x_{i}\right)\frac{l-i}{j-i},\left(i<l<j\right)$$

**Figure 2:**
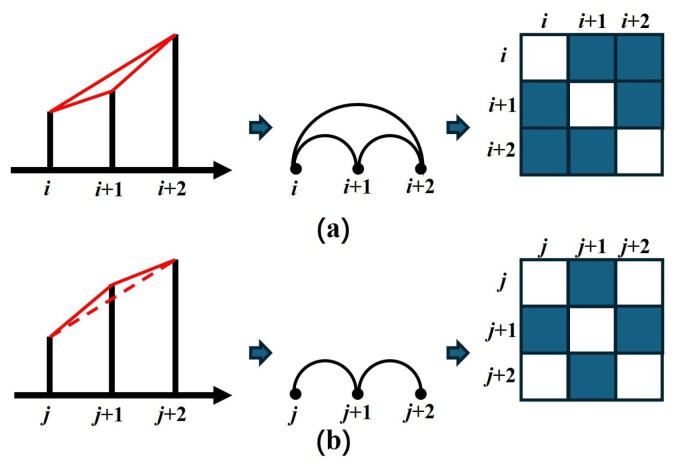
Time series, represented graphs, and its adjacency matrix. (**a**) The amplitude values at times *i* and i+2 satisfy the “visible relationship”, leading to the creation of an edge connecting nodes *i* and i+2 in the represented graph. (**b**) The amplitude values at times *j* and j+2 do not satisfy the “visible relationship”, hence the absence of an edge between nodes *j* and j+2.

Here, $x_{i}$ and $x_{j}$ denote the amplitude values at time points *i* and *j*, respectively, while $x_{l}$ represents the amplitude value at any intermediate time *l* between *i* and *j*. By identifying these visible relationships between amplitude values for all pairs of time points, the time series is transformed into a graph, effectively representing it in a high-dimensional space, as shown in Figure 2.

**Figure 3:**
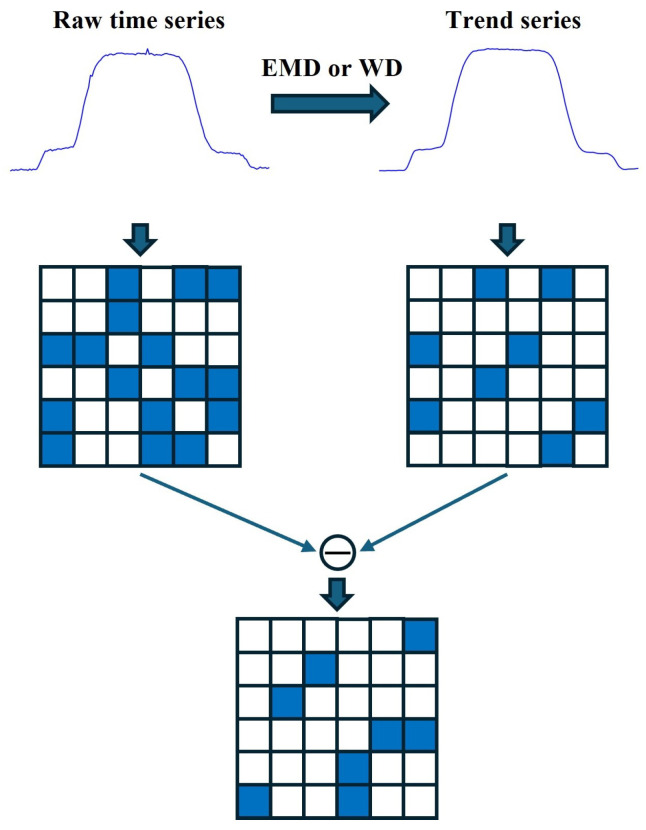
Steps to obtain differences caused by minor fluctuations in the representation space. Represent the raw time series and trend series separately as graphs, then calculate the topological differences of the graphs to represent the differences in representation space caused by minor fluctuations.

To enhance the identifiability of minor fluctuations, the raw time series and trend series are represented separately as graphs and then subtracted to quantify the differences induced by minor fluctuations in the representation space. The complete process for obtaining these differences is shown in Figure 3.

As illustrated in Figure 3, the presence of minor fluctuations in the raw time series can present a limitation. Specifically, the amplitude values near these minor fluctuations may not consistently satisfy the defined visible relationship criterion. Consequently, edges may be absent between corresponding nodes in the represented graph. Conversely, trend series devoid of such minor fluctuations do satisfy the visible relationship based on amplitude values at the defined time points, thus leading to the presence of edges between the corresponding nodes. The topological differences observed between these two graph representations, therefore, effectively capture information pertaining to the minor fluctuations present in the raw time series.

### 2.2. Further Feature Extraction for Difference Graph

**Figure 4:**
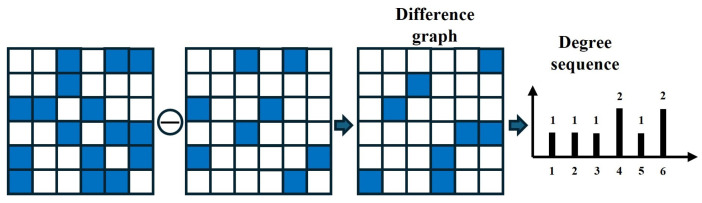
The degree sequence. By counting the degrees of nodes in the difference graph, a degree sequence is formed to extract the difference information caused by minor fluctuations.

Directly utilizing the difference graph as a classification feature is inappropriate because of the significant increase both space and time complexity. So, further feature extraction from the difference graph is essential to improve efficiency. Given that graph topology reflects differential information, and node degree quantifies the number of connections for each node, the degree sequence provides a concise representation of the difference graph’s topology. Therefore, we calculate the node degrees within the difference graph, construct a degree sequence, and extract relevant information, as depicted in Figure 4.

**Figure 5:**
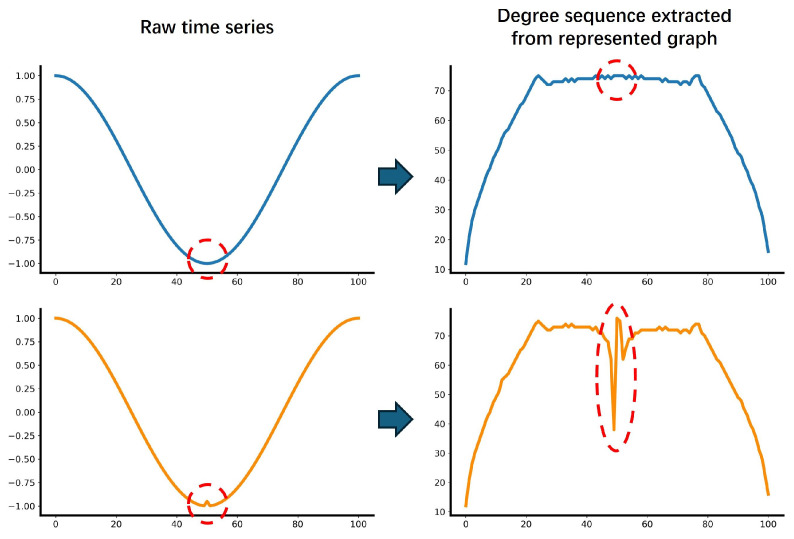
Example demonstration of further feature extraction effectiveness.

To demonstrate the efficacy of feature extraction from the difference graph in capturing minor fluctuations in time series, we present an illustrative example in Figure 5.

Figure 5 (left) displays the raw time series, where only minor fluctuations are apparent at the indicated locations. Figure 5 (right) presents the corresponding degree sequence derived from the nodes in the represented graph. As shown in Figure 5, the fluctuation disparity in the degree sequence is more pronounced than that observed in the raw time series. This suggests that our graph representation method makes minor fluctuations more distinguishable in the representation space, potentially leading to improved classifier accuracy.

Our approach extracts visible relational information from the amplitude values at each time point in the series, representing a relatively straightforward methodology. It obviates the need for pre-processing or transformation of the raw time series. In contrast, existing time series graph representation methods, such as Series2Graph [30], necessitate adaptive segmentation of the raw time series to identify recurring patterns. These methods treat each subsequence as a node in the graph and define edges based on the similarity between subsequences. While effective for capturing continuous sub-patterns within a time series, the Series2Graph method may exhibit limitations when dealing with minor fluctuations of short duration.

### 2.3. Optimization for Graph Representation Method and Complexity Analysis

**Figure 6:**
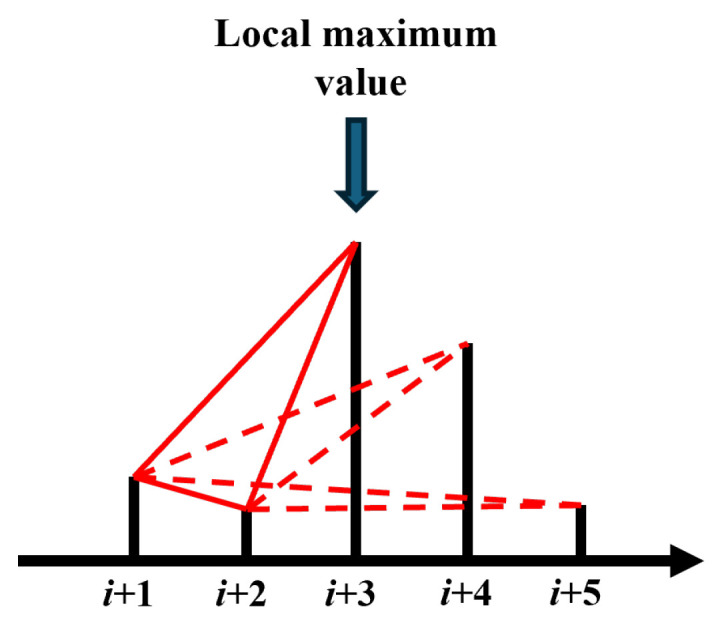
Local maximum value and visible relationship. A local maximum value appears at i+3, so the amplitudes at i+1 and i+2 do not satisfy the visible relationship with the amplitudes at i+4 and i+5.

The graph representation method described above exhibits relatively high computational complexity because it requires extracting the relationship between amplitudes across the time step. Specifically, given a time series of length *n*, the complexity is $O(n^{2})$. To enhance the applicability of this method across a wider range of resource-constrained sensor systems, optimization is necessary to reduce its computational burden. Examining the construction principle of this graph representation method, a key observation is that the “visible relationship” is not satisfied between amplitudes located before and after a local maximum, just as shown in Figure 6. Therefore, we can determine the visible relationships of each local maximum with all other data points. Subsequently, we can divide the series into subseries using the time of each local maximum as a delimiter and apply the graph representation method to each subseries independently.

**Figure 7:**
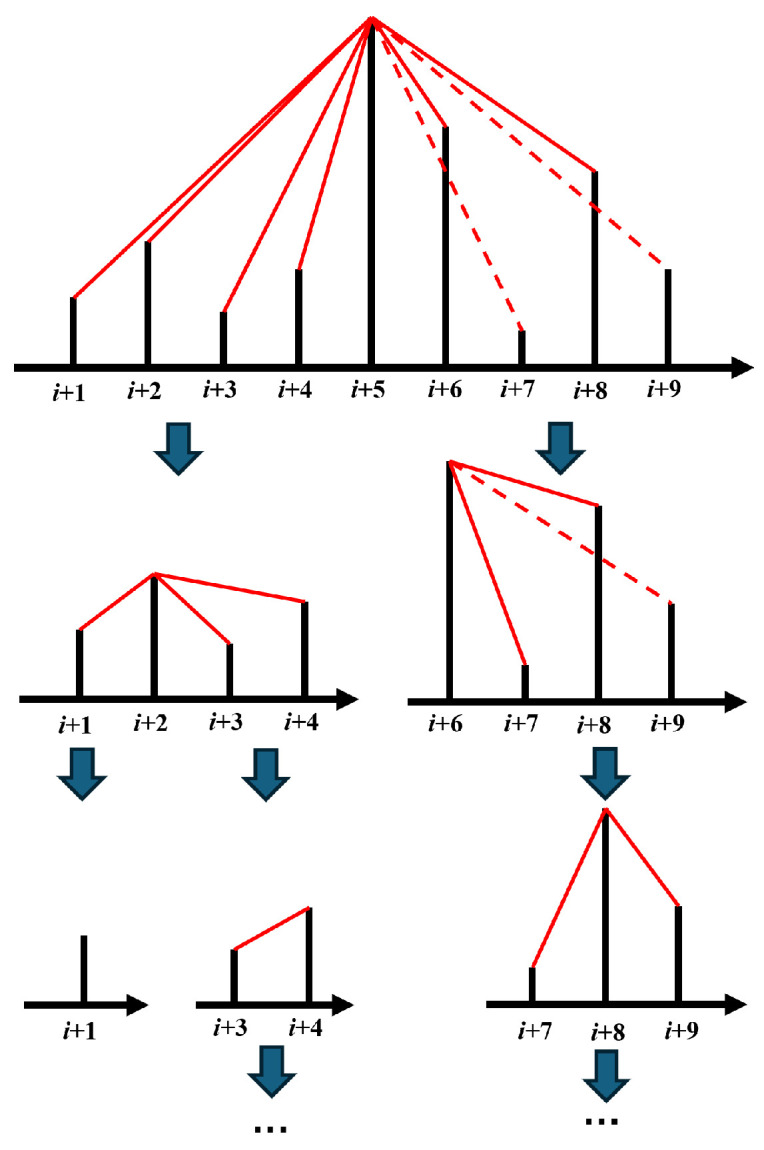
The optimized graph representation method based on the divide-and-conquer idea. Firstly, we get the maximum amplitude value (at time *i* + 5); then, determine the relationship between i+5 and other times; divide the time series into two subseries separated by i+5; and repeat the above steps in each subseries until the subseries cannot be further divided.

This approach extracts as much visible relationship information as possible while reducing the search space, thereby lowering the computational complexity. This optimized strategy aligns with the established principle of divide-and-conquer. The optimized representation method based on this divide-and-conquer approach is illustrated in Figure 7.

As shown in Figure 7, the local maximum value (at time i+5) is identified first. The visible relationship between the amplitude at time i+5 and the amplitude at all other times is then determined. Subsequently, the series is divided into two subseries, one spanning from time i+1 to i+4, and the other from time i+6 to i+9. The process is then applied to each subseries until further division is no longer possible.

For the optimized graph representation method, we conduct a complexity analysis. The computational complexity is related to the length of the time series. Let *n* denote the length of a time series, and let T(n) denote the average running time of the representation method for this time series. $T_{k}\left(n\right)$ represents the average running time for processing a subseries of length k+1 within the same framework. The relationship between these metrics can be expressed as follows:

(4) $$T\left(n\right)=\sum_{k=0}^{n-1}p\left(k+1\right)T_{k}\left(n\right)$$

where p(k+1) is the probability of dividing the time series into a subseries of length k+1. Since the probability of dividing the subseries is the same, and this probability is p(k+1)=1/n, Equation (4) can be expressed as

(5) $$T\left(n\right)=\sum_{k=0}^{n-1}\frac{1}{n}T_{k}\left(n\right)=\frac{1}{n}\sum_{k=0}^{n-1}\left(T\left(k\right)+T\left(n-k-1\right)+cn\right)$$

Further simplification leads to

(6) $$\frac{1}{n}(\sum_{k=0}^{n-1}\left(T\left(k\right)+\sum_{k=0}^{n-1}T\left(n-k-1\right)\right)+cn=\frac{2}{n}\sum_{k=0}^{n-1}T\left(k\right)+cn$$

To solve recursive equations,

(7) $$nT\left(n\right)=2\sum_{k=0}^{n-1}T\left(k\right)+cn^{2}$$

(8) $$\left(n-1\right)T\left(n-1\right)=2\sum_{k=0}^{n-1}T\left(k\right)+cn^{2}$$

Subtract Equations (7) and (8):

(9) nT(n)−(n−1)T(n−1)=2T(n−1)+c(2n−1)

(10) $$\frac{T(n)}{n+1}\leq \frac{T(n-1)}{n}+\frac{2c}{n}$$

We let

(11) $$G\left(n\right)=\frac{T(n)}{n+1}$$

Then

(12) $$\begin{array}{cc} G(n) & =G\left(n-1\right)+\frac{2c}{n}\\ \; & =G\left(n-2\right)+2c\left(\frac{1}{n-1}+\frac{1}{n}\right)\\ \; & =G\left(n-3\right)+2c\left(\frac{1}{n-2}+\frac{1}{n-1}+\frac{1}{n}\right)\\ \; & \cdots \\ \; & =G\left(n-k\right)+2c\left(\frac{1}{n-k+1}+\cdots +\frac{1}{n}\right) \end{array}$$

Then, we obtained

(13) $$G\left(1\right)+2c\sum_{k=0}^{n-2}\frac{1}{n-k}=2c\sum_{k=2}^{n}\frac{1}{k}\leq 2c*H_{n}\leq 2clogn$$

So, the average time complexity of the optimized graph representation method is

(14) T(n)=G(n)(n+1)=o(nlogn)

### 2.4. Mfsi-Tsc Framework

**Figure 8:**
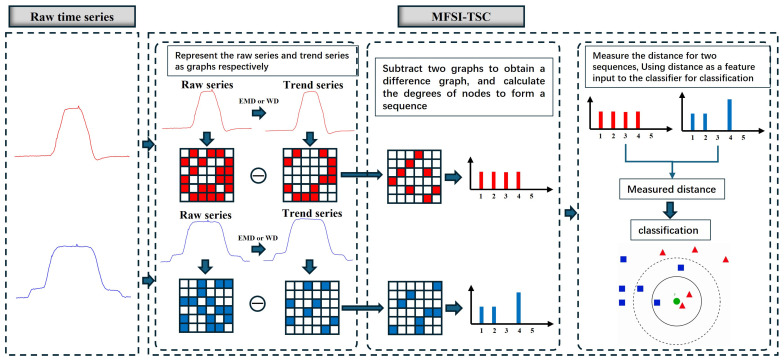
The MFSI-TSC framework utilizing the optimized graph representation.

Figure 8 illustrates the MFSI-TSC framework with the optimized graph representation method. This framework comprises several key components, as detailed below:

(1) First, MFSI-TSC extracts the trend series from the raw time series with either Empirical Mode Decomposition (EMD) or Wavelet Decomposition (WD). Subsequently, the optimized graph representation method is applied to both the raw time series and the extracted trend series. This process transforms both series into graph representations, achieved by extracting the visible relationships between the amplitude of each time point pair within the series. It represents a time series in a high-dimensional space.
(2) Second, MFSI-TSC extracts different information resulting from minor fluctuations in high-dimensional space. This is accomplished by subtracting the graph representing the raw time series from the graph representing the trend series, thereby revealing the topological differences attributable to the minor fluctuations. The degree of each node in the resulting difference graph is then obtained, producing a degree sequence that effectively represents the difference information stemming from minor fluctuations in a sequential format.
(3) Finally, the MFSI-TSC framework calculates the Euclidean distance between the degree sequences. This distance serves as a quantitative measure of the difference in minor fluctuations between the two series. This distance metric is then inputted as a feature into a conventional classifier for time series classification.

## 3. Experiment Results and Discussion

In this section, we conducted robustness and sensitivity analyses using synthetic data to examine the model’s behavior under controlled conditions. Then, ablation studies were performed on several publicly available datasets to quantify the contribution of each core module within MFSI-TSC. Finally, we evaluate the performance of our proposed MFSI-TSC framework on a real-world dataset and on datasets from the public UCR Time series Classification Archive [31]. We compare our method against several benchmark methods such as Euclidean Distance (ED) [32], Z-normalized Euclidean Distance (Z-norm) [33], Dynamic Time Warping (DTW) [34], Discrete Fourier Transform (DFT) [35], Word Extraction for Time Series Classification (WEASEL) [23], Bag-Of-SFA Symbols (BOSS) [36], Random Convolutional kernel Transform (ROCKET) [37], Shapelet Transforms (ST) [38], Series2Graph (S2G) [30], and Hierarchical Vote Collective of Transformation-based Ensembles (COTE) [29].

### 3.1. Robustness Analysis and Sensitivity Analysis

**Figure 9:**
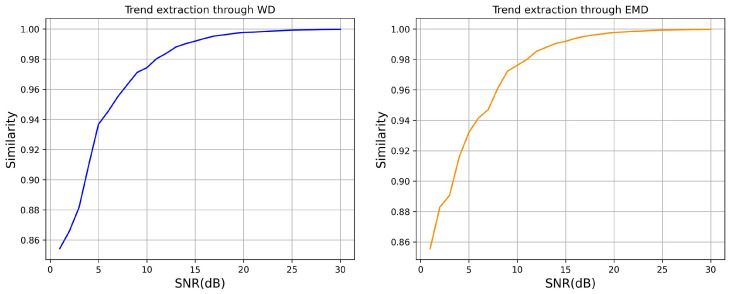
Robustness test results.

While EMD and WD were implemented within the MFSI-TSC framework to extract trend components from raw time series, their efficacy can be compromised when the scales of minor fluctuations and trends are similar. Under such conditions, EMD and WD may struggle to effectively disentangle these components. Consequently, we performed a robustness analysis to delineate the application limitations of the MFSI-TSC approach. This analysis involved generating a simulated trend series and superimposing random minor fluctuations upon it. Subsequently, EMD and WD were applied to decompose this composite time series. To quantify the accuracy of the decomposition, we measured the similarity between the extracted trend component and the original simulated trend. This allowed us to verify the point at which, as the signal-to-noise ratio (SNR) reaches a critical level, EMD and WD become unsuitable for reliably extracting minor fluctuation information. The results of this analysis are presented in Figure 9.

As illustrated in Figure 9, at a signal-to-noise ratio (SNR) of 1, the similarity between the trend decomposed by either EMD or WD and the original trend reaches approximately 86%. This suggests that the decomposed trends at this SNR level still retain some fluctuation information. Consequently, employing MFSI-TSC under such noisy conditions may lead to the overlooking of minor fluctuation features, thereby potentially diminishing classification accuracy. Conversely, when the SNR exceeds 20, the similarity between the decomposed and original trends surpasses 99%. Under these higher SNR conditions, the application of MFSI-TSC proves effective in discerning minor fluctuations in the data, ultimately contributing to improved classification accuracy.

**Figure 10:**
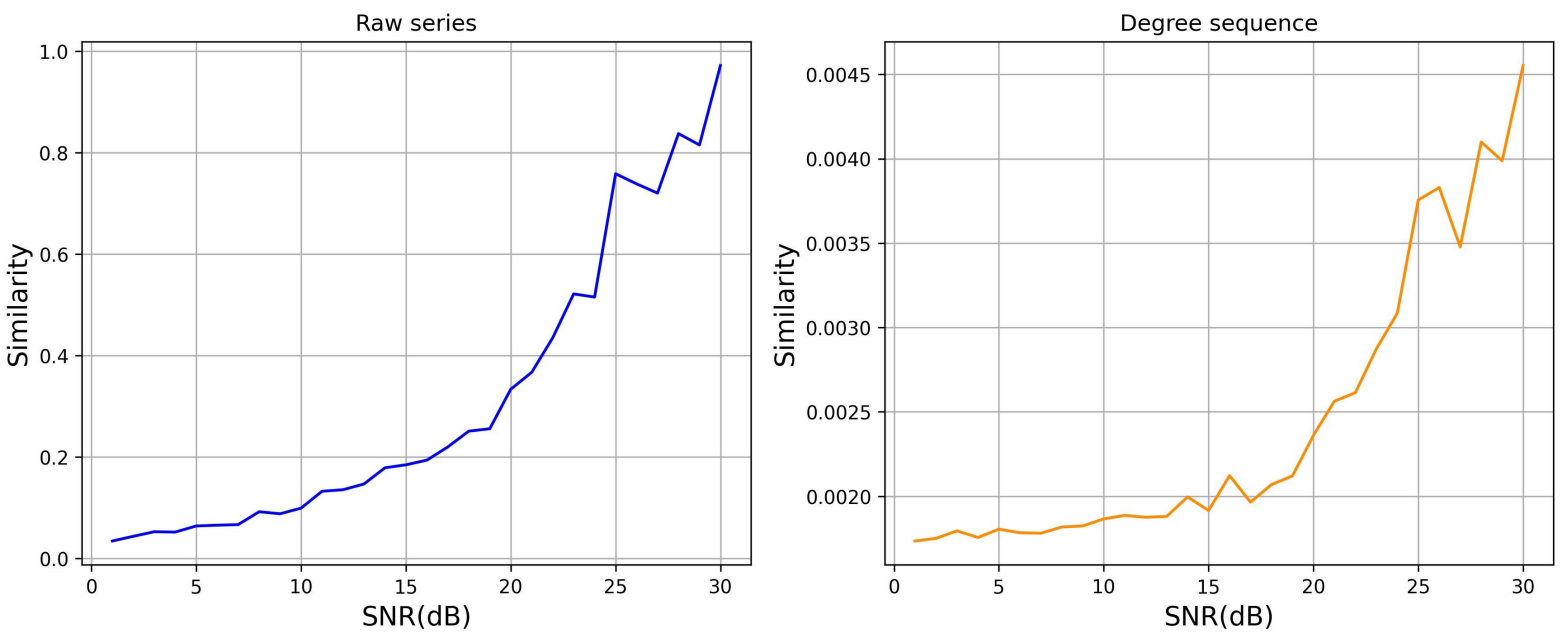
Sensitivity test results.

To assess the sensitivity of MFSI-TSC in detecting minor fluctuations, we performed a series of sensitivity analyses. We initially generated a synthetic time series and then introduced varying levels of noise to create a corresponding noisy time series. Subsequently, we applied MFSI-TSC to both the original and noisy time series to derive their respective degree sequences. We then quantified the similarity between both the raw time series and their corresponding degree sequences across a range of signal-to-noise ratios. The findings of this analysis are presented in Figure 10.

The observed similarity between the raw time series and its corresponding degree sequence exhibits a positive correlation with the SNR. Specifically, at SNR values exceeding 25, the similarity of the raw time series approaches 80%, while the similarity of the degree sequence derived via MFSI-TSC remains below 0.4%. This discrepancy illustrates the high sensitivity of MFSI-TSC to minor fluctuations within the time series. Even at an SNR of 30, where the raw time series similarity nears unity, the corresponding degree sequence similarity remains below 0.5%, further demonstrating MFSI-TSC’s capacity to effectively capture minor variations and, consequently, enhance classification accuracy.

### 3.2. Ablation Experiments

To further evaluate the contribution of each core module to overall classification performance, we conducted ablation experiments on five publicly available datasets. Specifically, we examined the impact of removing trend extraction (TE), visible relationship graph construction (VRGC), and topological difference quantification (TDQ) individually. The results of these ablation studies are summarized in Table 1.

As presented in Table 1, the trend extraction module exhibits a limited contribution to overall classification accuracy. This can be attributed to the separation of only minor fluctuation features from the raw time series. Despite this separation, the small amplitude of these fluctuations poses a challenge for the classifier to sensitively capture their inherent feature information. Similarly, the topology difference measurement module demonstrates a constrained impact on classification accuracy, although it significantly contributes to a reduction in complexity.

The graph construction module provides a notable enhancement to classification accuracy, as the resulting graph structure effectively reflects the small amplitude characteristics of minor fluctuations. Consequently, distinctions between these fluctuations can be discerned through analysis of the graph’s topology. Preceding graph construction with trend extraction from the raw time series amplifies the scale at which these minor fluctuations can be differentiated, leading to a further improvement in classification accuracy.

### 3.3. Performance Evaluation in a Real-World Dataset

This section presents an empirical evaluation of MFSI-TSC and ten benchmark methods using a real-world dataset obtained from China United Network Communications Group Co., Ltd. (Beijing, China). This dataset comprises sensor readings monitoring the operational status of base stations. Due to early minor faults in the power supply unit, the maximum operating voltage of the base station at full load was unstable, resulting in minor fluctuations. Subsequent manual validation confirmed the presence of 3 distinct types of incipient faults within the dataset. Both the training and testing sets consist of 310 instances, with each time series composed of 168 data points.

We evaluate the performance of MFSI-TSC and benchmark methods with the following six indicators. These indicators include Accuracy (Acc) [39], Adjusted Rand Index (ARI), Normalized Mutual Information (NMI) [40], Homogeneity (Hom) [41], Completeness (Com) [42], and V-Measure (V-mea) [43]. Accuracy is defined as the proportion of correctly classified samples to the total number of samples. It provides a general measure of classifier performance, with higher values indicating superior accuracy. To evaluate the alignment between classification results and truth labels, we employ the Adjusted Rand Index (ARI) and Normalized Mutual Information (NMI). Higher ARI and NMI values suggest greater concordance between the outcome and the truth labels. Furthermore, we assess the classification results using Homogeneity and Completeness scores. Homogeneity quantifies the extent to which each cluster contains only members of a single class, while Completeness measures the degree to which all members of a given class are assigned to the same cluster. The V-measure, representing the harmonic means of Homogeneity and Completeness, provides a balanced evaluation of the classing performance.

**Figure 11:**
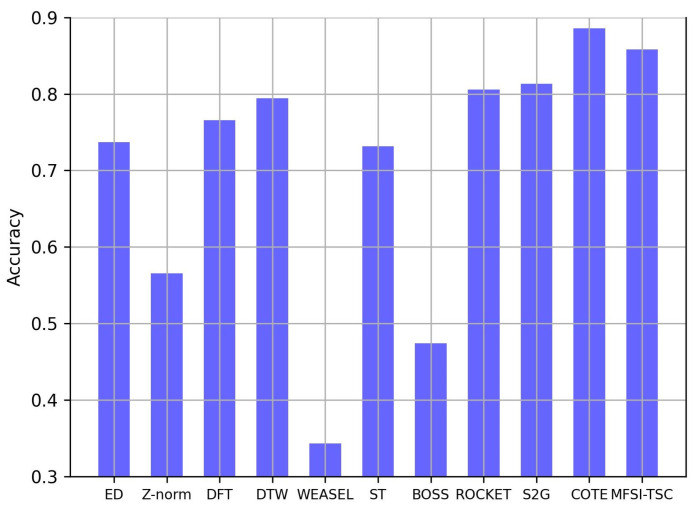
The accuracy of MFSI-TSC and other methods on a real-world dataset.

As shown in Table 2 and Figure 11, MFSI-TSC demonstrates superior classification performance compared with the other benchmark methods; it achieves an accuracy of 85%. The WEASEL method exhibits the poorest performance, due to its representation of time series as characters, smoothing the data and eliminating minor fluctuation features, which serve as the primary distinguishing feature. While BOSS also maps time series to discrete representations, its non-uniform amplitude mapping allows it to retain some minor fluctuation information, leading to a slightly improved classification accuracy compared with WEASEL.

Table 2 summarizes the evaluation metric of MFSI-TSC and other methods on a real-world dataset, while Figure 11 specifically visualizes their accuracy.

The accuracy of ED is higher than that of Z-norm, indicating that the standardization process of Z-norm may suppress the extraction of minor fluctuation features. The DFT method decomposes the data into low-frequency components representing trends and high-frequency components reflecting minor fluctuations. However, the amplitude of these minor fluctuations in the spectrum is often small and can be masked by the dominant trend components, leading to a lower accuracy than MFSI-TSC.

The ability of DTW to account for elastic scaling and align time series trends mitigates the interference of trend variations when identifying minor fluctuations. However, DTW still relies on calculating amplitude differences, which may diminish the significance of minor fluctuations for classification. The classification accuracy of DTW remains below 80%. ST aims to identify continuous subsequences within a time series as classification features and performs well when minor fluctuations occur consecutively. However, in this dataset, where minor fluctuations may appear at any time, the feature extraction of ST is often incomplete, resulting in a classification accuracy lower than even ED.

ROCKET utilizes randomly generated convolution kernels to extract various patterns and features from the time series. Due to the inherent limitations of random kernels in matching all possible series characteristics, its classification accuracy is slightly lower than that of MFSI-TSC. In contrast, MFSI-TSC represents time series as graphs and leverages topological differences induced by minor fluctuations to make them easier to distinguish. This approach allows MFSI-TSC to achieve the highest classification performance on this dataset, reaching an accuracy of 85%.

While Series2Graph (S2G) represents a recent advancement in time series representation by employing unsupervised learning to transform time series into graphs, it exhibits certain limitations. S2G adaptively decomposes the raw time series into subsequences, representing each subsequence as a node within a graph. Edge creation between nodes is contingent upon the similarity between the corresponding subsequences exceeding a pre-defined threshold. Although S2G proves effective in capturing large-scale fluctuation patterns within time series data, its performance is constrained when local, minor fluctuations constitute the primary distinguishing features for classification. Consequently, the classification accuracy achieved by S2G, at 81%, falls short of that attained by MFSI-TSC.

While the Hierarchical Vote Collective of Transformation-based Ensembles (COTE) method leverages deep learning to integrate multiple time series features, including spectral characteristics, time-frequency representations, and statistical data, to achieve high classification accuracy (reported as 88%), it suffers from significant limitations. Specifically, the pre-processing stage necessitates repeated feature extraction from the raw time series data prior to deep learning, resulting in substantial computational complexity. This high computational overhead renders COTE impractical for implementation in resource-constrained sensor systems.

**Figure 12:**
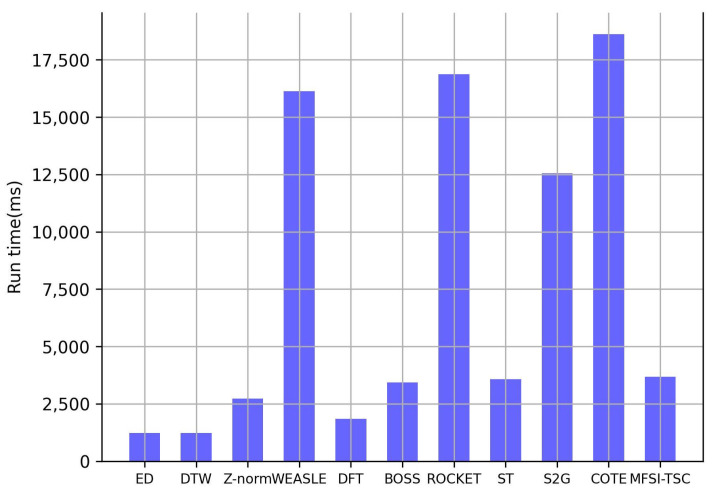
The runtime of MFSI-TSC and other methods on a real-world dataset.

The efficiency of MFSI-TSC and benchmark methods on the real-world dataset is illustrated in Figure 12. DTW and BOSS exhibit the longest runtimes. The path planning approach in DTW for matching across the entire time series results in high computational complexity. BOSS requires mapping each subsequence to a character and calculating inter-character distances with non-uniform amplitude division and subsequence segmentation, leading to a protracted execution time due to the multiple processing steps involved.

ED and Z-norm benefit from linear computational complexity and low algorithmic overhead, which directly classify raw time series based on Euclidean distance. The uniform division for the amplitude interval in WEASEL and the representation of time series as words enable fast computation. DFT maps time series to spectra and calculates the Euclidean distance between them. While the length of the spectra matches that of the raw time series, the availability of fast algorithms for DFT results in a runtime higher than ED but significantly lower than other methods.

ST needs to extract representative subsequences as classification features, requiring adjustments to the window size and search scope of the entire time series. However, its parallel search on raw time series leads to a running time only slightly higher than that of DFT. Similarly, the runtime of ROCKET is primarily determined by the convolution operation between the convolution kernel and the raw time series, resulting in a performance of running time comparable to that of ST. MFSI-TSC extracts visible relationships between amplitude at each time point in a time series, which is analogous to convolving the raw time series with a convolution kernel of length 1. Furthermore, our adoption of a divide-and-conquer approach to optimize the search for visible relationships effectively reduces the search space. Consequently, the runtime of MFSI-TSC is comparable to those of ST and ROCKET.

The S2G method exhibits a longer runtime than MFSI-TSC, which necessitates adaptive segmentation of the raw time series and subsequent similarity calculations between subsequences to establish edges between graph nodes. However, its runtime remains shorter in comparison to BOSS. This is because the BOSS needs to map each data point to a character one by one. COTE, by virtue of its requirement to extract multiple temporal features through continuous operations on the raw data, incurs the highest computational cost, with its runtime exceeding that of MFSI-TSC by several orders of magnitude.

### 3.4. Performance Evaluation in Public Datasets from UCR Time Series Classification Archive

This section presents an empirical evaluation of MFSI-TSC and eight benchmark methods using 55 datasets obtained from the public UCR Time series Classification Archive. The names of these datasets are shown in Table 3. In the MFSI-TSC framework, we deploy two conventional classifiers, SVM [44] and KNN [45]. For the SVM classifier, a radial basis function (RBF) kernel was employed with a regularization parameter set to 1. The KNN algorithm was implemented with the number of neighbors selected as 3.

Time series data within public datasets often exhibit similar trends, with variations arising primarily from minor fluctuations. Consider, for instance, electrocardiogram (ECG) datasets, where most human electrocardiogram signals share a common morphology. However, pathological conditions such as atrial enlargement can manifest as subtle abnormalities in the P wave of the ECG. Within the time series representation, these irregularities appear as minor fluctuations deviating from the established trend.

**Figure 13:**
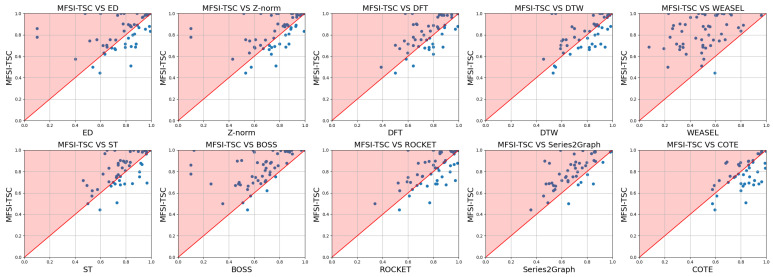
Comparison of accuracy between MFSI-TSC employed KNN and other methods.

Figure 13 presents scatter plots comparing the classification accuracy of MFSI-TSC against eight benchmark methods when employing a KNN classifier. In these plots, the horizontal axis denotes the accuracy of the benchmark method on a given dataset, while the vertical axis represents the corresponding accuracy achieved by MFSI-TSC. A diagonal line serves as a separator, with points above this separator line indicating that the datasets on which MFSI-TSC outperformed the benchmark method in terms of accuracy. The more points above the separator line, the better the classification accuracy of MFSI-TSC on more datasets.

**Figure 14:**
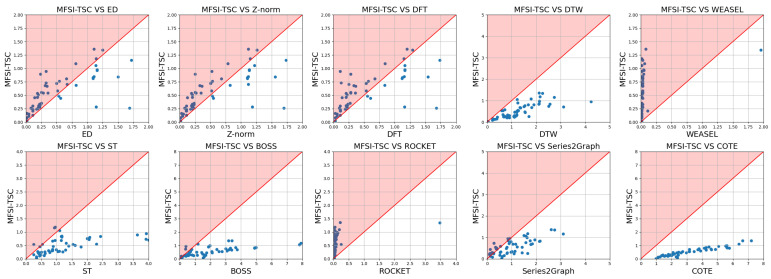
Comparison of runtime between MFSI-TSC employed KNN and other methods.

Figure 14 displays scatter plots contrasting the runtime of MFSI-TSC against the benchmark method. Points positioned above the diagonal indicate that the datasets on which MFSI-TSC required a longer runtime than the benchmark method. Thus, fewer points above the diagonal suggest greater efficiency of MFSI-TSC.

An inspection of the subfigures in Figure 13 comparing MFSI-TSC to ED and Z-norm reveals a similar distribution of points. This similarity stems from the fact that both ED and Z-norm are based on calculating Euclidean distance, with Z-norm standardizing the calculated ED distance. When minor fluctuations constitute the primary classification feature, the identification of the trend can overshadow the identification of these minor fluctuations, thereby limiting the classification accuracy of ED and Z-norm. Furthermore, the standardization performed by Z-norm on the ED distance may reduce the distinguishability of minor fluctuations, leading to a lower classification accuracy compared with ED.

In contrast, MFSI-TSC represents time series as graphs, capturing differences between time series through variations in graph topology. This approach makes minor fluctuations easier to distinguish, resulting in higher classification accuracy compared with ED and Z-norm. However, the efficiency of MFSI-TSC is not as favorable as that of ED and Z-norm, as evidenced by the many points above the diagonal in the respective subfigures of Figure 14. This indicates a greater computational complexity for MFSI-TSC. While ED and Z-norm employ linear distance calculations, MFSI-TSC needs to represent time series as graphs, which inherit the complexity that contributes to its longer runtime.

The subfigure in Figure 13 comparing MFSI-TSC and DFT demonstrates that most points lie above the diagonal, signifying superior accuracy for MFSI-TSC. DFT transforms time series from the time domain to the frequency domain, where low-frequency components represent the trend and high-frequency components reflect minor fluctuations. However, the amplitude of these high-frequency components is often low; the classifier still tends to prioritize learning the features of low-frequency components while ignoring the features of high-frequency components. The computational efficiency of MFSI-TSC and DFT is similar, with most points distributed around the diagonal. The complexity of optimized MFSI-TSC is o(nlogn); DFT also possesses a complexity of o(nlogn).

The subfigure in Figure 13 comparing MFSI-TSC and DTW is like the subfigure comparing MFSI-TSC and DFT. DTW addresses the challenge of elastic scaling by formulating it as a shortest path planning problem, identifying the optimal nonlinear alignment by determining the shortest path. Although DTW performs well in handling elastic stretching series, its classification accuracy remains low when minor fluctuations serve as the primary distinguishing feature. This limitation arises because DTW calculates differences based on raw amplitudes, where the trend can easily mask the more difference in minor fluctuations. Furthermore, the complexity of DTW is high, as illustrated in Figure 14, where all points are below the diagonal, indicating that MFSI-TSC possesses lower complexity than DTW.

The comparison between MFSI-TSC and WEASEL reveals a significant performance disparity. WEASEL exhibiting the poorest classification accuracy among the mentioned methods. Compared with WEASEL, BOSS achieves higher accuracy. Both WEASEL and BOSS are dictionary-based representation methods for the time series. WEASEL extracts feature vectors from time series using sliding windows and converts these vectors into words, effectively capturing linear translation characteristics. However, the uniformly amplitude segment strategy employed by WEASEL smooths out minor fluctuations, making the classifier insensitive to them. On the other hand, BOSS utilizes non-uniform amplitude segment strategy, rendering it more sensitive to minor fluctuations. However, BOSS still smooths out fluctuations, resulting in lower classification accuracy. Moreover, BOSS suffers from a long runtime due to the time-consuming mapping and representation of subseries using non-uniform amplitude partitioning, as shown in Figure 13.

The subfigure in Figure 13 comparing MFSI-TSC and ST indicates that MFSI-TSC provides better classification accuracy. While most points are above the diagonal, their proximity to the line suggests that the classification performance of ST and MFSI-TSC is comparable. ST extracts small subseries from time series as features, which may contain minor fluctuations. However, because these fluctuations can occur at any position and may not be continuous, the information captured by ST is incomplete. MFSI-TSC extracts all minor fluctuation information, leading to its higher classification accuracy. Nevertheless, MFSI-TSC exhibits a longer runtime than ST due to the time series representation and future feature extraction. ST directly searches on the raw time series to obtain subseries representing category features. Therefore, its complexity is lower than MFSI-TSC.

In the subfigure in Figure 13 comparing MFSI-TSC and ROCKET, most points are positioned above the diagonal, and MFSI-TSC still outperforms ROCKET. ROCKET generates random convolution kernels based on pre-defined parameters and then applies these kernels to the time series to extract various patterns and features. The pre-designed parameters result in low computational complexity. However, this approach can also lead to a mismatch between the convolution kernel and the specific characteristics of the time series, due to the poor generalization ability of these kernels. As a result, MFSI-TSC achieves higher classification accuracy. Due to the pre-designed nature of its convolution kernels, ROCKET exhibits a shorter runtime than MFSI-TSC, as illustrated in Figure 14.

As illustrated in Figure 13, most data points comparing MFSI-TSC and S2G lie above the diagonal, indicating the superior performance of MFSI-TSC. S2G operates by segmenting time series data and representing subsequences as nodes within a graph. Edge formation between these nodes is contingent upon the similarity between subsequences exceeding a predefined threshold. However, in the context of early fault diagnosis, the limited occurrence of minor fluctuations in the time series data often results in a highly connected graph structure with edges existing between most nodes. This over-connectivity compromises the discriminative power of the graph, thereby reducing the classification accuracy of the S2G method. Furthermore, the adaptive partitioning of molecular sequences inherent to the S2G approach necessitates multiple partitioning iterations, ultimately contributing to a longer algorithm execution time compared with MFSI-TSC.

While Figure 13 illustrates that COTE outperforms MFSI-TSC, as evidenced by most points falling below the diagonal, this performance differential is attributable to COTE’s comprehensive feature extraction approach. COTE leverages a diverse set of time series characteristics, encompassing spectral features, time-frequency representations, statistical properties, and shape attributes, thereby contributing to its enhanced accuracy and robustness. In contrast, MFSI-TSC relies on extracting visible relationship information, which consequently limits its classification performance relative to COTE. However, COTE’s extensive feature extraction process and subsequent deep network processing also result in a considerably longer execution time and increased computational resource demands compared with MFSI-TSC. This trade-off between performance and computational cost should be considered when selecting an appropriate time series classification method.

**Figure 15:**
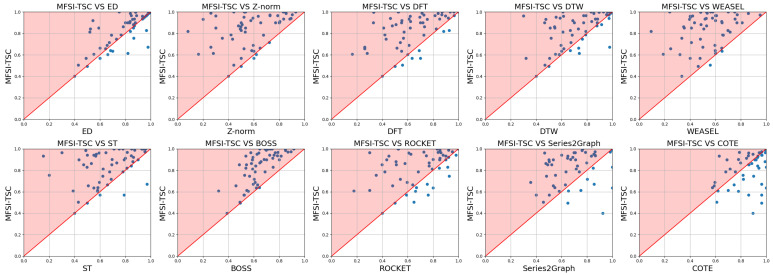
Comparison of accuracy between MFSI-TSC employed SVM and other methods.

**Figure 16:**
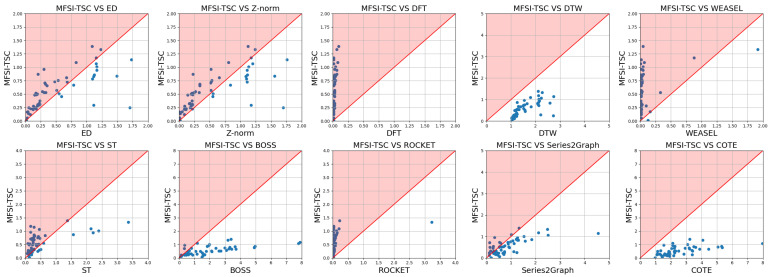
Comparison of runtime between MFSI-TSC employed SVM and other methods.

Figure 15 and Figure 16 present corresponding comparisons of accuracy and runtime for MFSI-TSC against the same benchmark methods but employing an SVM classifier. Consistent with the KNN-based MFSI-TSC framework, MFSI-TSC outperforms the comparative methods in terms of accuracy. In terms of efficiency, MFSI-TSC outperforms DTW and BOSS. The runtime of SVM-based MFSI-TSC is higher than that of KNN-based MFSI-TSC due to the non-linear nature of SVM classification, which demands greater computational resources.

**Figure 17:**
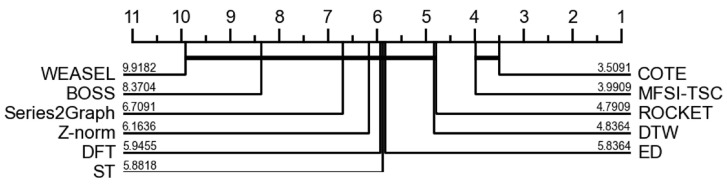
Critical Difference-diagram of MFSI-TSC employed KNN and other method.

To further evaluate the relative performance of the classification methods, a Nemenyi test was conducted on the classification accuracy data to determine critical differences. As illustrated in Figure 17, the critical difference values associated with COTE and MFSI-TSC when employing KNN are smaller than those of the remaining methods, suggesting a statistically significant enhancement in classification performance for these two approaches. Specifically, the critical difference for COTE is 3.5091, while for MFSI-TSC it is 3.9909. The observed difference between COTE and MFSI-TSC, however, is not statistically significant.

**Figure 18:**
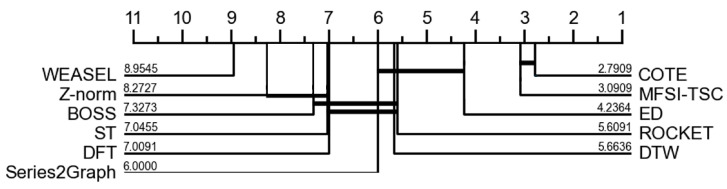
Critical Difference-diagram of MFSI-TSC employed SVM and other method.

Figure 18 presents the critical difference diagrams for MFSI-TSC utilizing SVM and other comparative methods. COTE achieves a critical difference of 2.7909, while MFSI-TSC attains a critical difference of 3.0909, demonstrating significantly superior performance compared with other algorithms. A second tier of classification methods, encompassing ED, ROCKET, Series2Graph, and DTW, exhibits an average critical difference of 5. These results indicate that COTE and MFSI-TSC employed SVMs have better classification performance than other benchmark methods. However, due to the high algorithm complexity of COTE, MFSI-TSC is more suitable for resource-constrained sensor systems.

The Wilcoxon test was employed to statistically evaluate the performance of MFSI-TSC against the comparative methods. The resulting *p*-values are presented in Table 4. As shown, the *p*-value obtained from the Wilcoxon test comparing MFSI-TSC and COTE exceeds the significance level of 0.05, suggesting that the performance difference between these two methods is not statistically significant. Conversely, the *p*-values derived from comparing MFSI-STC to the remaining methods are substantially below 0.05, indicating statistically significant performance differences. Considering the considerably greater computational complexity of COTE relative to MFSI-TSC, the adoption of MFSI-STC represents a more practical and efficient approach for early fault diagnosis in sensor systems.

## 4. Conclusions

This paper introduces MFSI-TSC, a novel time series classification framework based on graph representation to enhance the distinguishability for minor fluctuations. MFSI-TSC initially employs Empirical Mode Decomposition (EMD) or Wavelet Decomposition (WD) to extract the trend from raw time series. Subsequently, both the raw time series and the extracted trend series are represented as graphs. The framework then quantifies the topological difference between these two graphs, effectively capturing high-dimensional disparities induced by minor fluctuations. Specifically, the degree of each node within the difference graph is extracted, forming a degree sequence that is utilized as a feature vector for a classifier. By incorporating visible relationship information during feature extraction and representing time series as high-dimensional graphs, MFSI-TSC makes minor fluctuations easier to identify in time series classification. Consequently, the MFSI-TSC framework demonstrates strong classification performance, particularly when classifying time series where minor fluctuations serve as the primary distinguishing feature.

A limitation of the MFSI-TSC stems from its reliance on EMD and WD for extracting trend information from time series. When the magnitude of minor fluctuations approaches that of the trends, EMD and WD may struggle to effectively differentiate these components, potentially reducing classification accuracy. Consequently, MFSI-TSC is best suited for scenarios where the power spectral density of the time series is comparatively high. While EMD and WD offer valuable tools for MFSI-TSC, their efficacy in extracting trends from highly complex data can be limited. Further research is therefore warranted to identify and evaluate alternative methodologies capable of robust trend extraction across a wider spectrum of time series complexities. Furthermore, the limited utilization of real-world datasets within this study constitutes a potential limitation. The discrepancies may exist between the research findings and the performance observed in practical applications. Building upon MFSI-TSC’s graph-based representation, future research could explore graph-based time series prediction methods to mitigate the phase shift issues often encountered in traditional prediction techniques.

## Figures and Tables

**Table 1:** The results of the ablation experiment.

Method	ACSF1	CBF	PowerCons	DodgerLoopDay	TwoLeadECG
KNN	0.6333	0.6	0.5375	0.5143	0.5778
KNN+TE	0.6667	0.65	0.5469	0.5498	0.6353
KNN+VRGC+TDQ	0.7846	0.8	0.6478	0.6844	0.8337
KNN+TE+VRGC	0.7976	1	0.6534	0.7138	0.9667
KNN+TE+VRGC+TDQ	0.8112	1	0.6725	0.7456	0.9889
SVM	0.6667	0.69	0.5435	0.5459	0.7
SVM+TE	0.6889	0.72	0.6254	0.6333	0.7517
SVM+VRGC+TDQ	0.8667	0.93	0.7045	0.7143	0.9458
SVM+TE+VRGC	0.9177	1	0.7122	0.7278	0.9667
SVM+TE+VRGC+TDQ	0.9177	1	0.7395	0.7554	0.9889

**Table 2:** Evaluation metrics of MFSI-TSC and other methods in real datasets.

	ED	Z-Norm	DFT	DTW	WEASEL	ST	BOSS	ROCKET	S2G	COTE	MFSI-TSC
Acc	0.73226	0.56129	0.76129	0.79032	0.34193	0.73548	0.47096	0.80322	0.81322	0.88562	0.85806
ARI	0.37686	0.11844	0.40992	0.47469	0.00287	0.35188	0.03901	0.49919	0.50186	0.60254	0.58572
NMI	0.35214	0.16542	0.41711	0.43435	0.02560	0.35187	0.08855	0.45265	0.46895	0.59351	0.53051
Hom	0.35198	0.17275	0.41819	0.43293	0.02612	0.35270	0.09487	0.45138	0.47182	0.60158	0.52889
Com	0.35231	0.15868	0.41604	0.43577	0.02511	0.35104	0.08301	0.45392	0.47225	0.60579	0.53214
V-mea	0.35214	0.16542	0.41711	0.43435	0.02560	0.35187	0.08855	0.45265	0.46935	0.61524	0.53051

**Table 3:** The name of public datasets from the UCR Time series Classification Archive.

Dataset
ACSF1 Beef BME Car CBF Rock UMD Fish Plane ArrowHead
BeetleFly BirdChicken Chinatown Coffee DiatomSizeReduction
DistalPhalanxOutlineAgeGroup DistalPhalanxTW
DodgerLoopDay DodgerLoopGame DodgerLoopWeekend
ECG200 ECGFiveDays FaceFour Fungi GunPoint
GunPointAgeSpan GunPointMaleVersusFemale
GunPointOldVersusYoung Ham Herring HouseTwenty
InsectEPGSmallTrain ItalyPowerDemand Lightning2 Lightning7
Meat MoteStrain OliveOil PickupGestureWiimoteZ
PowerCons ProximalPhalanxOutlineAgeGroup
ShakeGestureWiimoteZ ShapeletSim SmoothSubspace
SonyAIBORobotSurface1 SonyAIBORobotSurface2 Symbols
SyntheticControl ToeSegmentation1 ToeSegmentation2 Trace
TwoLeadECG Wine Worms WormsTwoClass

**Table 4:** The *p*-value in Wilcoxon test.

	ED	DTW	Z-Norm	WEASLE	DFT	BOSS	ROCKET	ST	S2G	COTE
MFSI-TSC (KNN)	$1.69*10^{-2}$	$1.58*10^{-3}$	$1.09*10^{-10}$	$4.81*10^{-12}$	$2.11*10^{-7}$	$2.36*10^{-8}$	$4.73*10^{-4}$	$3.37*10^{-6}$	$2.89*10^{-6}$	$3.59*10^{-1}$
MFSI-TSC (SVM)	$1.89*10^{-2}$	$6.32*10^{-4}$	$2.29*10^{-10}$	$1.58*10^{-12}$	$5.18*10^{-7}$	$1.39*10^{-8}$	$5.85*10^{-4}$	$3.69*10^{-6}$	$3.87*10^{-6}$	$2.68*10^{-1}$

## Data Availability

Publicly available datasets were analyzed in this study.
